# *In Vitro* Evaluation of Novel Inhibitors against the NS2B-NS3 Protease of Dengue Fever Virus Type 4

**DOI:** 10.3390/molecules181215600

**Published:** 2013-12-13

**Authors:** Thi Thanh Hanh Nguyen, Sun Lee, Hsi-Kai Wang, Hsin-Yen Chen, Ying-Ta Wu, Simon C. Lin, Do-Won Kim, Doman Kim

**Affiliations:** 1 Department of Biotechnology and Bioengineering, Chonnam National University, 77 Yongbong-ro, Buk-gu, Gwangju 500-757, Korea; E-Mails: nguyenhanh040981@yahoo.com (T.T.H.N.); mockerysn@naver.com (S.L.); 2 Research Institute of Bio Food Industry, The Green Bio Research Complex, Seoul National University, San 967-2 Shin-ri, Pyeongchang-gun, Gangwon-do 232-916, Korea; 3 Research Center for Information Technology Innovation, Academia Sinica, 128, Sec.2, Academia Rd., Nankang, Taipei 11529, Taiwan; E-Mails: hsikai.wang@twgrid.org (H.-K.W.); hychen@twgrid.org (H.-Y.C.); Simon.Lin@cern.ch (S.C.L.); 4 Genomics Research Center, Academia Sinica, 128, Sec.2, Academia Rd., Nankang, Taipei 11529, Taiwan; E-Mail: ywu@gate.sinica.edu.tw; 5 Department of Physics, Gangneung-Wonju National University, Gangneung 210-702, Korea; E-Mail: dwkim@gwnu.ac.kr

**Keywords:** dengue fever, inhibitors, NS2B-NS3 protease, virtual screening

## Abstract

The discovery of potent therapeutic compounds against dengue virus is urgently needed. The NS2B-NS3 protease (NS2B-NS3^pro^) of dengue fever virus carries out all enzymatic activities needed for polyprotein processing and is considered to be amenable to antiviral inhibition by analogy. Virtual screening of 300,000 compounds using Autodock 3 on the GVSS platform was conducted to identify novel inhibitors against the NS2B-NS3^pro^. Thirty-six compounds were selected for *in vitro* assay against NS2B-NS3^pro^ expressed in *Pichia pastoris*. Seven novel compounds were identified as inhibitors with *IC_50_* values of 3.9 ± 0.6–86.7 ± 3.6 μM. Three strong NS2B-NS3^pro^ inhibitors were further confirmed as competitive inhibitors with *K_i_* values of 4.0 ± 0.4, 4.9 ± 0.3, and 3.4 ± 0.1 μM, respectively. Hydrophobic and hydrogen bond interactions between amino acid residues in the NS3^pro^ active site with inhibition compounds were also identified.

## 1. Introduction

Among members of the *Flavivirus* genus dengue virus (DENV) is responsible for the highest rate of disease and mortality and consists of a group of four serologically related viruses referred to as DENV types 1–4. Infection with these viruses results in a range of clinical diseases such as dengue fever, dengue hemorrhagic fever, and dengue shock syndrome [1,2,3]. Global epidemics of DENV have occurred over the past few years. There were 390 million (95% credible interval 284–528 million infections) dengue infections per year, of which 96 million (67–136 million infections) apparently manifested some level of disease severity [4]. DENV are transmitted to humans by the bite of infective female mosquitoes of the genus *Aedes*.

DENV has a single-stranded RNA genome that is packaged by the virus capsid protein in a host-derived lipid bilayer and is surrounded by 180 copies of two glycoproteins. The complete virion is approximately 50 nm in diameter and contains an approximate 10.7 kb positive-sensed RNA genome that has one open reading frame encoding a single polyprotein [2]. The 5'-end encodes three structural proteins: the capsid (C); membrane precursor protein (prM) proteolytically cleaved by the host protease furin to form the membrane protein in mature virions; and the envelope (E), constituting the enveloped virus particle [2,5]. Seven non-structural (NS) proteins essential for viral replication are encoded by the remainder of the genome. The order of proteins encoded is 5'-C-prM-E-NS1-NS2A-NS2B-NS3-NS4A-NS4B-NS5-3' [5,6]. NS3^pro^ is responsible for cleaving both in the *cis* and *trans* directions to generate viral proteins that are essential for viral replication and maturation of infectious dengue virions. A number of different strategies have been employed to search for DENV NS2B-NS3 protease inhibitors, including high-throughput screening [7,8,9], synthesizing rationally designed substrate-based peptidomimetics [10,11], cyclopeptide [12], and structure-based virtual screening (SBVS) [13,14,15] as well as screening natural products [16,17]. However, only a few peptide or small molecule inhibitors of the DENV NS2B-NS3 protease with moderate activity have so far been reported [18,19]. There are currently no effective antiviral agents to treat dengue infection [20] as well as no licensed vaccine against dengue infection is available, and the most advanced dengue vaccine candidate did not meet expectation in a recent large trial [4,21,22].

Over the past decade, high-throughput virtual screening (VS), and particularly substrate-based virtual screening (SBVS) has emerged as a reliable, cost-effective and time-saving technique for discovering lead compounds as an alternative to high-throughput screening [23]. VS, as applied to new enzyme inhibitor discovery, involves docking, computational fitting of compound structures to the enzyme active site, and scoring and ranking of each compound [24,25]. Grid Application Platform Virtual Screening Service (GVSS) has been developed with the Autodock 3.0.5 docking engine [26]. Thus, a user friendly grid service, GVSS, was developed to conduct large-scale molecular docking easily [26].

In this study, NS2B-NS3^pro^ was expressed in *Pichia pastoris* culture. This enzyme was used for *in vitro* enzyme inhibition assays against 36 compounds selected from VS of NS3^pro^ using the GVSS. The novel compound inhibitors were tested for their *IC_50_* values and used for an enzyme kinetic study. 

## 2. Results and Discussion

### 2.1. Expression of Active NS2B-NS3^pro^ in P. pastoris

**Figure 1:**
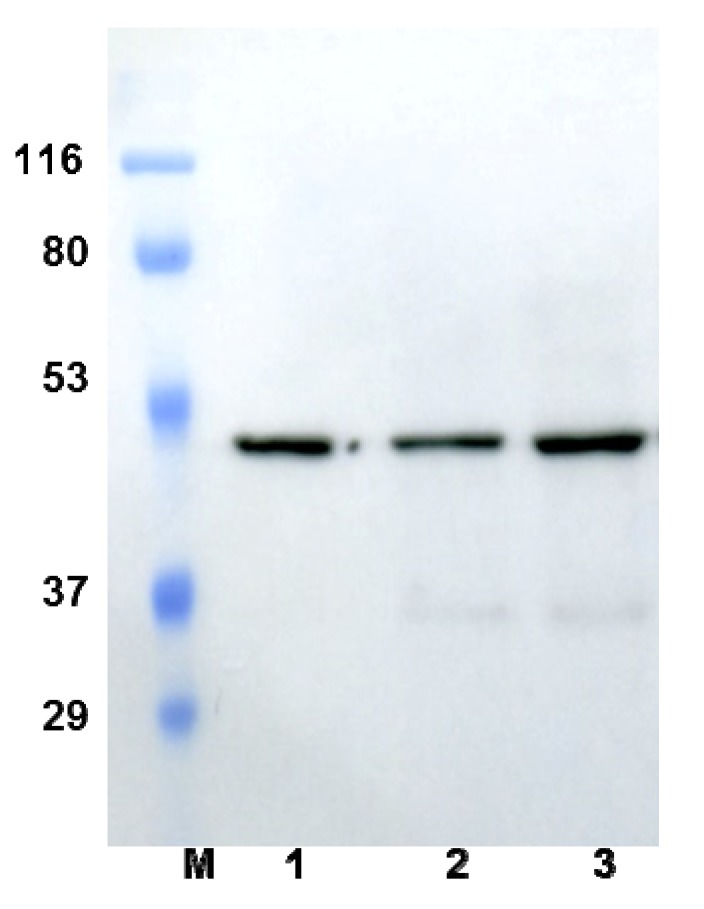
Western blot results of recombinant NS2B-NS3^pro^ after 48, 72, and 96 h of expression. Lane M, markers; lanes 1, 2, and 3, western blots conducted after 48, 72, and 96 h expression, respectively.

DENV4 NS2B-NS3^pro^, fused to the α-factor secretion signal sequence and placed under the control of the methanol inducible alcohol oxidase promoter, was constructed to express the secreted protein in *P. pastoris*. The expressed NS2B-NS3^pro^ was 45 kDa based on western blot using the anti-His antibody (Figure 1). The NS2B-NS3^pro^ proteolytic activity was monitored with fluorogenic tetrapeptide substrate containing the 7-amino-4-methylcoumarin (AMC) leaving group (benzoyl-norleucine-lysine-arginine-arginine-7-amino-4-methylcoumarin, Bachem, Bubendorf, Switzerland) for an increase in fluorescence at a λ_ex_ = 380 nm and λ_em_ = 460 nm at 37 °C. No enzyme activity was detected before induction or in the *P. pastoris* negative control without NS2B-NS3^pro^ gene (data not shown). NS2B-NS3^pro^ activity increased steadily with increasing culture time (and induction time) and reached a final activity of 480 U·L^−1^ after 96 h of induction with methanol. 

NS2B-NS3^pro^ was purified from *P. pastoris* culture supernatants. After dialysis to remove the potassium phosphate buffer, 77.6 mg of proteins remained in the culture medium supernatant. A total of 9.7 mg of NS2B-NS3^pro^ was isolated by ammonium sulfate precipitation. The final preparation of NS2B-NS3^pro^ was 1.4 mg following ion-exchange chromatography using a DEAE-Sepharose column, which resulted in a 13.5-fold purification (Table 1). The recombinant protein was expressed in approximately 0.8 mg·L^−1^ of the induced culture medium and specific activity of the purified recombinant NS2B-NS3^pro^ was 59.3 U·mg^−1^. The Michaelis–Menten constant (*K_m_*) derived with AMC peptide substrates from the Lineweaver–Burk plot was 1.60 ± 0.1 μM (Figure S1). 

**Table 1:** Purification summary of NS2B-NS3^pro^ in *P. pastoris*.

Purification Step	Total Volume (mL)	Total Protein (mg)	Total Activity (U)	Specific Activity (U/mg)	Purification (fold)	Yield (%)
Crude enzyme	1850	200.2	888.5	4.4	1	100
10 kDa cut-off	800	77.6	807.8	10.4	2.4	90.9
Ammonium sulfate precipitation	31.5	9.7	567.0	58.5	13.3	63.8
DEAE-Sepharose	5.0	1.4	83.0	59.3	13.5	9.3

### 2.2. GVSS against NS3^pro^

GVSS was developed with the Autodock 3.0.5 docking engine [26]. A JAVA-based graphical user interface and Grid Application Platform (GAP) allows end-users to specify target and compound libraries, set up docking parameters, monitor docking jobs and computing resources, visualize and refine docking results, and download the final results. GVSS was designed for conducting large-scale molecular docking more easily by providing a user-friendly grid service [26]. A total of 300,000 compounds were used for the large-scale screening with GVSS against NS3^pro^ protein (PDB accession number 2VBC [27]) by using 4,167 CPU per day, and the results were completed in 60 days. A top scoring of 10% was selected from the first post-docking filtering strategy based on the free binding energy of the lowest energy conformation. Thirty-six compounds were selected for *in vitro* assay based on their free energy binding (Table S1) and H-bonds interactions with amino acid residues at the NS3^pro^ active site. 

### 2.3. *In Vitro* Assay for NS2B-NS3^pro^ Potential Inhibiting Activity

Each compound obtained after VS was tested in duplicate at 100 μM for its ability to inhibit NS2B-NS3^pro^ activity. The primary inhibition assay of the 36 selected compounds is shown in Table S2. Among them, seven compounds that showed higher inhibition activities against NS2B-NS3^pro^ were selected for determining their *IC_50_* values (Table 2). Their physicochemical properties were shown in Table S2. The chemical structure of each compound is depicted in Table 2. The seven compounds displayed NS2B-NS3^pro^ inhibitory activities with *IC_50_* values of 3.9 ± 0.6–86.7 ± 3.6 μM (Table 2). 

**Table 2:** Inhibitory activities of the identified compounds against recombinant NS2B-NS3^pro^.

Compound	Chemdiv ID	Chemical Structure	Free Binding Energy (kcal·mol^−1^)	Inhibition ^a^ (%)	*IC_50_* (μM)
**2**	6049-2540	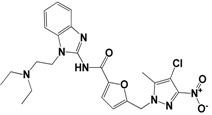	−13.2	95.2	15.6 ± 0.9
**5**	E881-0223	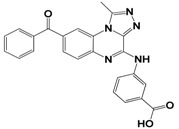	−10.3	58.8	86.7 ± 3.6
**12**	3011-0208	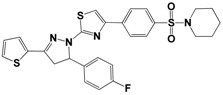	−11.3	85.2	32.2 ± 1.9
**14**	G642-2349	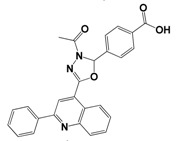	−10.4	98.2	12.5 ± 0.5
**22**	K286-0036	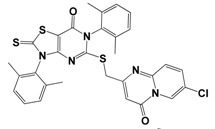	−11.9	91.7	3.9 ± 0.6
**27**	C090-0497	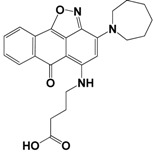	−11.3	82.5	26.4 ± 2.6
**29**	F575-0314	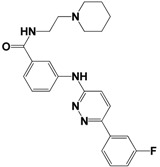	−11.8	66.6	75.3 ± 1.9

^a^ Inhibition activity at 100 μM by using 1.65 µM AMC peptide substrate.

**Figure 2:**
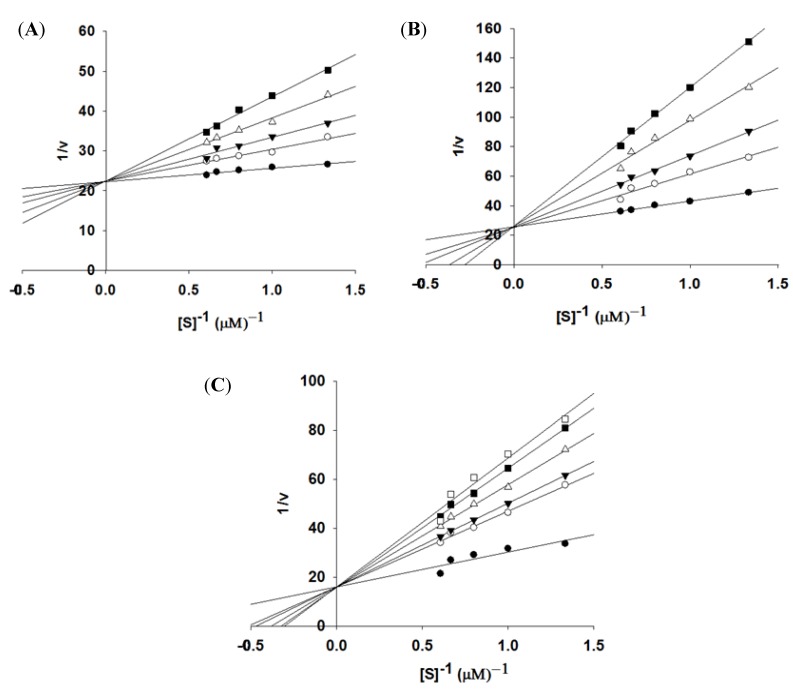
Lineweaver–Burk plot analyses for inhibition of compounds **2**, **14**, and **22** against NS2B-NS3^pro^. (**A**): compound **2** concentration 0 μM (●), 5 μM (○), 10 μM (▼), 15 μM (∆), and 20 μM (■); (**B**): compound **14** concentration 0 μM (●), 7.5 μM (○), 15 μM (▼), 20 μM (∆), and 25 μM (■); (**C**): compound **22** concentration 0 μM (●), 1 μM (○), 2 μM (▼), 3 μM (∆), 4.5 μM (■), and 6 μM (□).

Compounds **2**, **14**, and **22**, which displayed over 90% inhibition activity at 100 μM, were subjected to kinetic characterization. Inhibitory kinetic experiments were performed at different constant inhibitor concentrations and different substrate concentrations. Lineweaver–Burk and Dixon plots were used to analyze the inhibition modes of these compounds. *K_i_* values were calculated from the Dixon plots. The slopes of the lines confirmed that compounds **2**, **14**, and **22** were competitive inhibitors against recombinant NS2B-NS3^pro^ (Figure 2A–C) using AMC peptide substrates. Based on the linear regression analysis of the Dixon plot (Figure S2 A–C), the inhibitor constants (*K*_i_) were 4.0 ± 0.4, 4.9 ± 0.3, and 3.4 ± 0.1 μM for compounds **2**, **14**, and **22**, respectively. 

**Figure 3:**
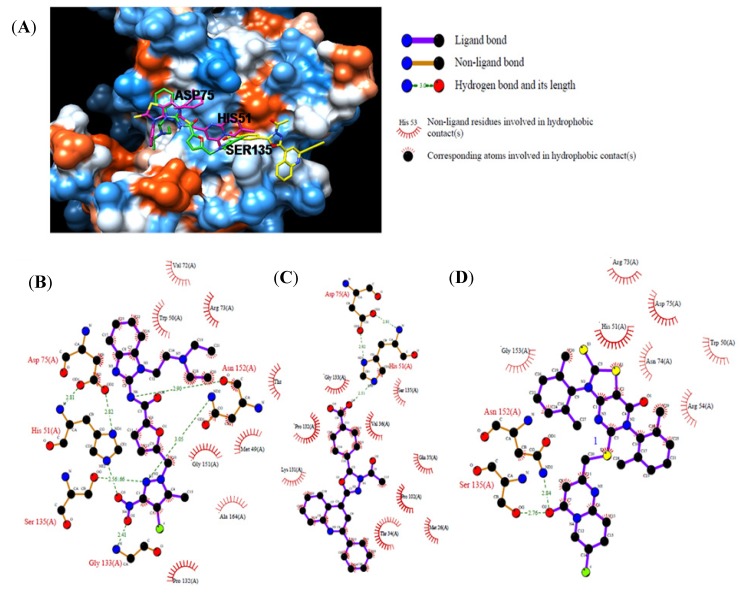
Computational docking and hydrophobic and H-bond interactions of compounds **2**, **14**, and **22** with residues in the NS3^pro^ active-site pocket. (**A**): comparison of binding modes of compounds **2** (green), **14** (yellow), and **22** (magenta) at the NS3^pro^ active-site pocket. (**B**), (**C**), (**D**): hydrophobic and H-bond interactions of compounds **2**, **14**, and **22** with amino acid residues in the NS3^pro^ active site, respectively. H-bond interactions are represented by green dashed lines.

Compounds **2**, **14**, and **22** were analyzed and compared by molecular docking as potent binders to the NS3^pro^ active site pocket (Figure 3A). Figure 3B provides the details of the specific interactions between compound **2** and NS3^pro^; carbon atoms of compound **2** formed hydrophobic interactions with Met49, Trp50, Val72, Arg73, Asp75, Pro132, Gly151, Asn152, Ala164, and Thr166 of NS3^pro^. The O^2^and O^3^atom of nitro group of compound 2 accepted hydrogen bond (H-bond) from amino group of Gly133 and N atom of the imidazole group of His 51 at 2.41 and 2.56 Å, respectively. The N^1^ atom of pyrazol group of compound **2** donated two H-bonds: one was the Ser135 hydroxyl group at 2.66 Å and the other was the Asn152 amine group at 3.05 Å. The N^6^ atom of amine group of compound **2** had one H-bond with the Asn152 carboxyl group at 2.90 Å. Figure 3C provides the details of the specific interactions between compound **14** and NS3^pro^; carbon atoms of compound **14** formed hydrophobic interactions with Met26, Thr34, Gln35, Val36, Pro102, Lys131, Pro132, Gly133, and Ser135 of NS3^pro^. The O^4^ atom of benzoic acid group of compound **14** had two H-bond interactions: one H-bond with the carboxyl group of Val136at 3.13 Å and the other with N atom of imidazole group of His51 at 2.51 Å. Figure 3D provides the details of the specific interactions between compound **22** and NS3^pro^; The carbon atoms of compound **22** formed hydrophobic interactions with Trp50, His51, Arg54, Arg73, Asn74, Asp75, Asn152, and Gly153. The O^2^ atom of pyrimidine group of compound **22** had two H-bonds: one with the hydroxyl group of Ser135 at 2.76 Å and the other with amine group of Asn152 at 2.84 Å. Among the three compounds, compounds **2** and **14** had enhanced two special H-bond interactions between His51 and Asp75 of NS3^pro^: the N atom amino group and imidazole group of His51 donated with O atom of second carboxyl group of the Asp75 at 2.82 and 2.81 Å, respectively (Figure 3B,C).

## 3. Experimental

### 3.1. Preparation of Recombinant DENV4 NS2B-NS3^pro^

The NS2B-NS3^pro^ gene was synthesized for *P. pastoris* after codon optimalization (DNA2.0, Menlo Park, CA, USA) based on the amino acid sequence of NS2B-NS3^pro^ (AMBL AAW30973.1) [28]. The protein encoding the NS2B-NS3^pro^ comprises 49 NS2B amino acid residues (amino acid residues 1393–1441), which are linked by a flexible GGGGSGGGG linker with the 186 NS3^pro^ amino acid residues (amino acid residues 1475–1660) (Figure S3). The NS2B-NS3pro gene (730 bp) was isolated from the vector (pJ201) with the NS2B-NS3^pro^ gene by cutting with *EcoRI/NotI* (Takara, Shiga, Japan) and was ligated into the *EcoRI/NotI*-digested pPICZαA vector (Invitrogen, Carlsbad, CA, USA). This construct allowed secretion of NS3^pro^ into the culture medium because of the presence of an in frame N-terminal α-factor secretion signal peptide. The novel construct was named pPICZαA-NS2B-NS3^pro^ and transformed into *E. coli* DH5α (Invitrogen) using a standard heat shock method. Transformants harboring pPICZαA-NS2B-NS3^pro^ were selected from low salt medium LB agar [1.0% (w/v) tryptone, 0.5% (w/v) yeast extract, 0.5% (w/v) NaCl, pH 7.5] containing 25 μg·mL^−1^ ZeocinTM (Invitrogen). The pPICZαA-NS2B-NS3pro plasmid was amplified in *E. coli* DH5α, linearized by *SacI* digestion, and transformed into *P. pastoris* GS115 using a modified lithium chloride method. The pPICZαA vector was also linearized by *SacI* digestion and transformed into *P. pastoris* GS115 as a negative control strain. After transformation, cells were plated on YPD agar [1% (w/v) yeast extract, 2% (w/v) peptone, and 2% (w/v) glucose] containing 100 µg Zeocin·mL^−1^ and incubated for 3 days at 30 °C. Positives were screened by polymerase chain reaction (PCR) with two sets of primers (Bioneer, Deajeon, Korea): set one contained the NS2B-NS3pro primers [DV4-F (5'-GAATTCGCTGATCTTA GTTTAG-3') and DV4-R (5'-GCGGCCGCTTTCTTTCTAAA-3')] and set two contained the α-factor and 3AOX1 primers. 

The selected clone was inoculated for gene expression into 100 mL BMGY [1% (w/v) yeast extract, 2% (w/v) peptone, 100 mM potassium phosphate, pH 6.0, 1.34% (w/v) yeast nitrogen base, 4 × 10−5% (w/v) biotin, and 1% glycerol] in a 500 mL flask and incubated at 30 °C with shaking at 250 rpm for 16 h. The cells were harvested and resuspended in 500 mL BMMY [1% (w/v) yeast extract, 2% (w/v) peptone, 100 mM potassium phosphate, pH 6.0, 1.34% (v/v) yeast nitrogen base, 4 × 10−5% (w/v) biotin, and 0.5% (v/v) methanol] medium in a 2 L flask to an optical density at 600 nm of 1.0 and then incubated at 30 °C with shaking at 250 rpm. To maintain methanol induction by the AOX1 promoter, 0.5% (v/v) methanol was fed every 24 h during the fermentation period [29]. Culture aliquots (1 mL each) were collected every 24 h for analyses of NS2B-NS3^pro^ expression and enzyme activity. pPICZαA-transformed *P. pastoris* X-33 proteins were expressed as a negative control. 

The yeast cells were then separated from the broth by centrifugation at 8,000 *×g* for 15 min at 4 °C. The pellet was discarded, and the supernatant was exchanged with 20 mM Tris buffer (pH 7.5) using a 10 kDa membrane (Millipore, Billerica, MA, USA). The supernatant was used for ammonium sulfate fractionation (from 0%–90%). The proteins obtained were dissolved in 20 mM Tris buffer (pH 7.5) and dialyzed against 20 mM Tris buffer (pH 7.5). Concentrated protein sample was loaded onto a DEAE-Sepharose column equilibrated with a low-salt buffer containing 50 mM NaCl and 40 mM Tris-HCl (pH 7.5). The recombinant protein was eluted with a salt gradient of 50–1,000 mM NaCl in a buffer containing 40 mM Tris-HCl (pH 7.5). One mL fractions were collected and analyzed by sodium dodecyl sulfate-polyacrylamide gel electrophoresis (SDS-PAGE) and checked for enzyme activity. The fractions containing recombinant NS2B-NS3pro were pooled and concentrated using a 9,000 Da molecular weight cut-off Pierce^®^ concentrator. 

NS2B-NS3^pro^ proteolytic activity was measured in a SpectraMax Gemini XPS apparatus (Molecular Devices, Sunnyvale, CA, USA) (λex = 380 nm, λem = 460 nm) performed in a final volume of 100 µL containing 40 mM Tris-HCl (pH 7.5) and 25 µM of fluorogenic tetrapeptide substrate, benzoyl-norleucine-lysine-arginine-arginine-7-amino-4-methyl coumarin (Bachem, Bubendorf, Switzerland), at 37 °C using a fluorescence plate reader. The proteolytic reaction was monitored by an increase in fluorescence (relative fluorescence units min-1), which was subsequently converted to µM·min^−1^ from a standard 7-amino-4-methyl coumarin (AMC) calibration curve [30]. One unit of NS2B-NS3^pro^ activity was defined as the quantity of enzyme required to produce 1 μM of AMC per min at 37 °C and pH 7.5 in 40 mM Tris-HCl. 

NS2B-NS3^pro^ kinetic parameters were obtained using 1–20 μM AMC peptide substrates in the fluorescent assay with a 20 min measurement period. Reaction responses were linear within this time period. The Michaelis–Menten constant (*K_m_*) was calculated from a Lineweaver–Burk using the SigmaPlot program (Systat Software, San Diego, CA, USA).

### 3.2. Virtual Screening

The VS process for DENV4 has been described previously [26]. The three-dimensional coordinates in the X-ray crystal structure of NS3^pro^ (PDB accession number 2VBC) [27] obtained from the Protein Data Bank [26,31] were used as the receptor model for the structural-based VS docking simulations. The NS3^pro^ docking library comprised 300,000 compounds from ChemDiv Inc. (San Diego, CA, USA), a commercially available compound library, was used for VS. AutoDock version 3.0.5 was used for the computational molecular docking simulation of flexible small molecules to rigid proteins using ligand and rigid proteins [32,33]. Large-scale computations were conducted between 2VBC and 300,000 compounds using the GVSS [26]. Important docking parameters for the Lamarckian genetic algorithm were a population size of 100 individuals, maximum of 1.5 million energy evaluations, maximum of 27,000 generations, mutation rate of 0.02, crossover rate of 0.8, and 50 docking runs (each docking job produced 50 docked conformations). The probability of performing a local search on an individual in the population was set to 0.06 and the maximum number of iterations per local search was set to 300. One percentage top scoring function was extracted and the potential hydrogen bond among ligands and key residues in the active site pocket of HMA was identified using chimera software [34], and selected compounds for next step were analyzed for their hydrophobic and H-bond interactions using the Ligplot program [35]. Among 300,000 compounds, 36 were selected for testing *in vitro* inhibitory activity against NS2B-NS3^pro^ based on hydrogen bond interactions [25,36].

### 3.3. Inhibition Assay

Each compound was tested in duplicate at a concentration of 100 μM for its ability to inhibit NS2B-NS3^pro^ activity during initial screening. Each compound was dissolved in dimethyl sulfoxide (DMSO) as a 5 mM stock solution. Assays were performed in a reaction mixture (final volume 100 μL) containing 0.04 U enzymes, 1.65 μM AMC peptide substrate, 100 μM of inhibitor, and 40 mM Tris buffer, pH 7.5. Reactions were run for 20 min at 37 °C with continuous fluorescence monitoring using a SpectraMax Gemini XPS apparatus (Molecular Devices, Eugene, OR, USA) with excitation and fluorescence emission wavelengths of 380 nm and 460 nm, respectively. 

### 3.4. Enzyme Kinetics

Inhibitor kinetic studies were performed for compounds **2**, **14**, and **22** against NS2B-NS3^pro^. The NS2B-NS3^pro^ kinetic parameters were obtained using 0.75, 1, 1.25, 1.5, and 1.65 μM AMC peptide substrate in the fluorescent assay with a 20 min measurement period. Reaction responses were linear within this time period. The inhibitor concentration used was 0–20 μM for compound **2**, 0–25 μM for compound **14**, and 0–6 μM for compound **22**. The type of inhibition was determined using Lineweaver–Burk plots, and *K_i_* values were determined with a Dixon plot (1/v as a function of [I], inhibitor concentration) from different inhibitor concentrations [36,37]. 

## 4. Conclusions

Thirty-six compounds from VS were tested for their inhibitory activities towards NS2B-NS3^pro^ expressed from *P. pastoris*. The *IC_50_* values of seven were 3.9 ± 0.6–86.7 ± 3.6 μM, and three of the compounds (**2**, **14**, and **22**) were competitive inhibitors of NS2B-NS3^pro^ with *K_i_* values of 4.0 ± 0.4, 4.9 ± 0.3, and 3.4 ± 0.1 μM, respectively. Detailed docking simulation binding mode analyses showed that the inhibitors were stabilized by formation of H-bonds with catalytic residues and the establishment of hydrophobic interactions with amino acids in the NS3^pro^ active site pocket. More detailed inhibition studies are underway to elucidate the inhibitory mechanism of compounds **2**, **14**, and **22**.

## Supplementary Materials

Supplementary materials can be accessed at: http://www.mdpi.com/1420-3049/18/12/15600/s1.
