# Supplementary material for: In Vitro Evaluation of Novel Inhibitors against the NS2B-NS3 Protease of Dengue Fever Virus Type 4
Source: Molecules. 2013 Dec 13;18(12):15600–12. doi: 10.3390/molecules181215600 (PMC6269914; doi:10.3390/molecules181215600)

Supplementary Materials for

In Vitro Evaluation of Novel Inhibitors Against the NS2B-NS3 Protease of Dengue Fever Virus Type 4

Table S1. Free binding energy and inhibition activities of the identified compounds against recombinant NS2B-NS3^{pro}.

Compound No.	Chemdiv ID [a]	Chemical Name	Free Binding Energy (kcal. mol^{-1})	Inhibition Activity [b] (%)
**1**	2036-0800	4-(2,6-bis(3-hydroxyphenyl)-1,3,5,7-tetraoxo-1,2,3,5,6,7-hexahydropyrrolo[3,4-f]isoindole-4-carbonyl)benzoic acid	−12.14	15.05
**2**	6049-2540	5-((4-chloro-5-methyl-3-nitro-1H-pyrazol-1-yl)methyl)-N-(1-(2-(diethylamino)ethyl)-1H-benzo[d]imidazol-2-yl)furan-2-carboxamide	−13.23	95.23
**3**	C684-0059	1-(4-(2,5-dimethylphenyl)piperazin-1-yl)-4-(10-methylbis([1,2,4]triazolo)[4,3-a:1',5'-c]quinazolin-3-yl)butan-1-one	−11.34	2.61
**4**	8007-4601	2,6-bis(1,3-dioxo-2-phenylisoindolin-5-yl)pyrrolo[3,4-f]isoindole-1,3,5,7(2H,6H)-tetraone	−10.95	4.64
**5**	E881-0223	3-((8-benzoyl-1-methyl-[1,2,4]triazolo[4,3-a]quinoxalin-4-yl)amino)benzoic acid	−10.32	58.83
**6**	K785-0146	(5,7-dimethyl-6-(3-methylbenzyl)pyrazolo[1,5-a]pyrimidin-3-yl)(4-(2,5-dimethylphenyl)piperazin-1-yl)methanone	−11.22	8.61
**7**	2688-0025	N-(2-benzoyl-4-bromophenyl)-3-((6-bromo-4-phenylquinazolin-2-yl)amino)benzamide	−10.68	21.31
**8**	E626-0966	N-(3-(piperidin-1-yl)propyl)-1-(6-(p-tolyl)imidazo[2,1-b][1,3,4]thiadiazol-2-yl)piperidine-3-carboxamide	−12.40	33.48
**9**	K979-0542	N-cycloheptyl-3-(3-methoxyphenyl)-4-oxo-3,4-dihydrophthalazine-1-carboxamide	−9.87	23.33
**10**	8009-4947	[1,1'-biphenyl]-4,4'-diylbis((3-amino-4,5,6-trimethylthieno[2,3-b]pyridin-2-yl)methanone)	−12.55	7.59
**11**	D008-0060	4-((4aR,5R,5aR,8aR,9S)-10-(4-(tert-butyl)phenyl)-2,6,8-trioxo-2,3,4a,5,5a,6,8a,9,9a,10-decahydro-5,9-methanothiazolo[5',4':5,6]thiopyrano[2,3-f]isoindol-7(8H)-yl)butanoic acid	−10.77	12.46
**12**	3011-0208	2-(5-(4-fluorophenyl)-3-(thiophen-2-yl)-4,5-dihydro-1H-pyrazol-1-yl)-4-(4-(piperidin-1-ylsulfonyl)phenyl)thiazole	−11.27	85.17
**13**	8011-5845	5-(5,6-dibromo-1,3-dioxohexahydro-1H-4,7-methanoisoindol-2(3H)-yl)-2-morpholinobenzoic acid	−10.79	5.55
**14**	G642-2349	4-(3-acetyl-5-(2-phenylquinolin-4-yl)-2,3-dihydro-1,3,4-oxadiazol-2-yl)benzoic acid	−10.42	98.15

Table S1. *Cont.*

Compound No.	Chemdiv ID [a]	Chemical Name	Free Binding Energy (kcal. mol^{-1})	Inhibition Activity [b] (%)
**15**	G608-0283	2-(4-((5-methyl-2-(m-tolyl)-[1,2,4]triazolo[1,5-a]pyrimidin-7-yl)amino)phenyl)acetic acid	−11.32	17.36
**16**	8004-3490	2-(1-isobutyl-5,5-dimethyl-3-(naphthalen-1-yl)-2-oxoimidazolidin-4-yl)-4-(naphthalen-1-yl)-1,2,4-oxadiazolidine-3,5-dione	−12.07	33.28
**17**	D052-0041	3-(((1-methyl-1H-tetrazol-5-yl)thio)methyl)-7-(2-(5-methyl-4-nitro-1H-pyrazol-1-yl)acetamido)-8-oxo-5-thia-1-azabicyclo[4.2.0]oct-2-ene-2-carboxylic acid	−13.23	23.01
**18**	E017-0021	1-(5-(4-(4-(5-chloro-2-methylphenyl)piperazine-1-carbonyl)piperidin-1-yl)-1,3,4-thiadiazol-2-yl)pyrrolidin-2-one	−10.26	0.15
**19**	D385-0151	methyl 2-((1-(4,6-di(piperidin-1-yl)-1,3,5-triazin-2-yl)-1H-1,2,4-triazol-3-yl)thio)acetate	−10.96	10.28
**20**	K953-0293	N-(4-((2-(benzo[d][1,3]dioxol-5-yl)-1,3-dioxoisoindolin-5-yl)oxy)phenyl)-1-oxo-4-phenyl-1H-isochromene-3-carboxamide	−12.94	28.87
**21**	8005-2734	2-(3-(2-(3,5-diphenyl-4,5-dihydro-1H-pyrazol-1-yl)thiazol-4-yl)phenyl)isoindoline-1,3-dione	−11.11	21.27
**22**	K286-0036	5-[[(7-chloro-4-oxo-4H-pyrido[1,2-a]pyrimidin-2-yl)methyl)sulfanyl)-3,6-bis(2,6-dimethylphenyl)-2-thioxo-2,3-dihydro[1,3]thiazolo[4,5-d]pyrimidin-7(6H)-one	−11.85	91.65
**23**	G397-0661	N-(3-(4-cyclohexylpiperazin-1-yl)propyl)-1-(5-(2-oxopyrrolidin-1-yl)-1,3,4-thiadiazol-2-yl)piperidine-3-carboxamide	−11.46	17.71
**24**	G426-0201	3-(3,7-dimethyl-6-oxo-1-phenyl-6,7-dihydro-1H-pyrazolo[3,4-b]pyrazin-5-yl)-N-(2-(4-methylpiperazin-1-yl)ethyl)propanamide	−12.57	29.46
**25**	5692-1397	4,4'-(1,1'-(1,4-phenylenebis(methylene))bis(1H-benzo[d]imidazole-2,1-diyl))bis(1,2,5-oxadiazol-3-amine)	−11.2	36.45
**26**	D308-0105	N-(1,5-dimethyl-3-oxo-2-phenyl-2,3-dihydro-1H-pyrazol-4-yl)-2-((5,9-dimethyl-[1,2,4]triazolo[4,3-a]quinolin-1-yl)thio)acetamide	−12.23	28.11
**27**	C090-0497	4-((3-(azepan-1-yl)-6-oxo-6H-anthra[1,9-cd]isoxazol-5-yl)amino)butanoic acid	−11.34	82.53
**28**	K823-2046	N-cycloheptyl-2,5-dimethyl-4-(4-(pyridin-2-yl)piperazin-1-yl)thieno[2,3-d]pyrimidine-6-carboxamide	−11.21	32.84

Table S1. *Cont.*

Compound No.	Chemdiv ID [a]	Chemical Name	Free Binding Energy (kcal. mol^{-1})	Inhibition Activity [b] (%)
**29**	F575-0314	3-((6-(3-fluorophenyl)pyridazin-3-yl)amino)-N-(2-(piperidin-1-yl)ethyl)benzamide	−11.75	66.55
**30**	E017-0513	N-(3-(4-ethylpiperazin-1-yl)propyl)-1-(5-(2-oxopyrrolidin-1-yl)-1,3,4-thiadiazol-2-yl)piperidine-3-carboxamide	−11.85	1.3
**31**	8016-9443	N^2,N^2-dimethyl-6-(((4-methyl-5-((2-methyl-5-nitro-1H-imidazol-1-yl)methyl)-4H-1,2,4-triazol-3-yl)thio)methyl)-1,3,5-triazine-2,4-diamine	−12.31	6.49
**32**	6228-1590	N-(1-benzyl-1H-benzo[d]imidazol-2-yl)-5-((3-nitro-1H-1,2,4-triazol-1-yl)methyl)furan-2-carboxamide	−12.39	45.55
**33**	4486-0033	(E)-1,4-bis((5,6-diphenyl-1,2,4-triazin-3-yl)thio)but-2-ene	−12.77	40.52
**34**	6193-0962	2-((4aR,5R,5aR,8aR,9S)-2,6,8-trioxo-3,10-diphenyl-2,3,4a,5,5a,6,8a,9,9a,10-decahydro-5,9-methanothiazolo[5',4':5,6]thiopyrano[2,3-f]isoindol-7(8H)-yl)acetic acid	−10.64	39.67
**35**	K284-2326	3,3'-(ethane-1,2-diyl)bis(2-(((7-chloro-4-oxo-4H-pyrido[1,2-a]pyrimidin-2-yl)methyl)thio)quinazolin-4(3H)-one)	−13.42	48.66
**36**	3952-0694	2-(3-fluorophenyl)-7-((2-(3-fluorophenyl)-4-oxo-4H-benzo[d][1,3]oxazin-6-yl)methyl)-4H-benzo[d][1,3]oxazin-4-one	−10.54	34.33

[a] http://eu.chemdiv.com; [b] Inhibition activity (%) after primary inhibition assay at a 100 µM concentration of inhibitors.

Table S2. Physicochemical properties of 7 hit compounds obtained after virtual screening.

Compounds	Chemdiv ID	Log P *	MW	H-Donors	H-Acceptors
**2**	6049-2540	4.66	499.17	1	8
**5**	E881-0223	4.68	423.13	2	6
**12**	3011-0208	6.27	552.11	0	8
**14**	G642-2349	4.66	437.14	1	6
**22**	K286-0036	7.84	617.08	0	8
**27**	C090-0497	4.23	419.18	2	5
**29**	F575-0314	4.72	419.51	2	5

* The logarithm of the ratio of the concentrations of the un-ionized solute in the solvents is called log P. The log P value is also known as a measure of lipophilicity.

Figure S1. Lineweaver–Burk plot to determine the NS2B-NS3pro K_m value. The reaction was conducted at various substrate concentrations to obtain the K_m value of the enzyme. Sigma plot was used to fit the kinetic data using Lineweaver–Burk double reciprocal plots.

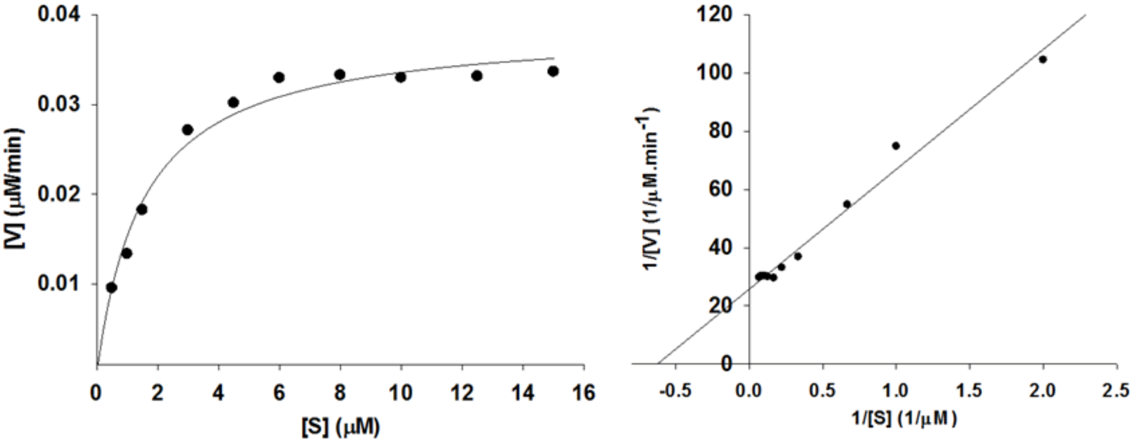

Figure S2. Dixon plot analyses for the inhibition of NS2B-NS3pro by compounds **2**, **14**, and **22**. The kinetic constants, K_i, were calculated using linear regression analysis. **A**, **B**, **C**: AMC peptide substrate concentration 0.75 μM (●), 1 μM (○), 1.25 μM (▼), 1.5 μM (Δ), and 1.65 μM (■).

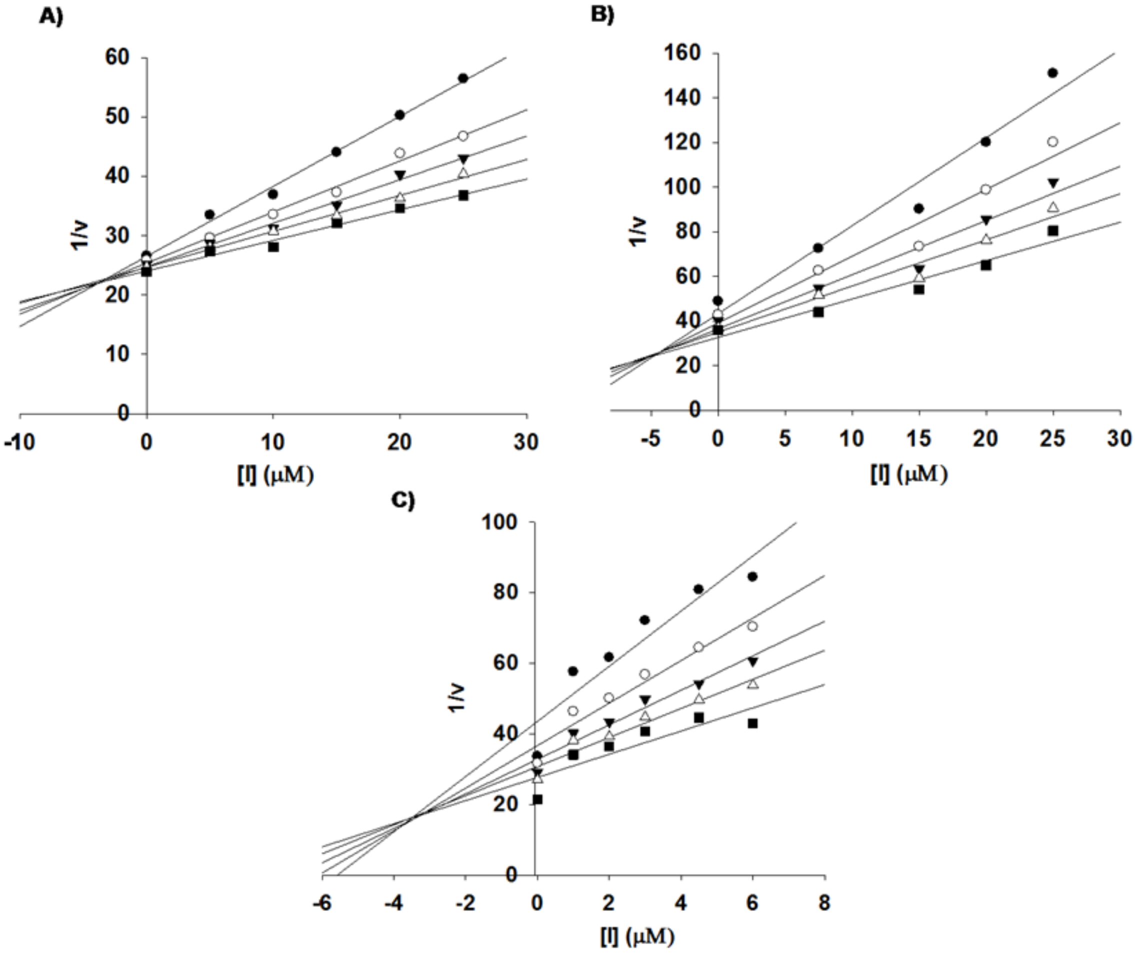

Figure S3. Nucleotide sequence of the NS2B-NS3^{pro} gene. The codon optimized gene was synthesized based on the amino acid sequence of AMBL AAW30973.1.

```
1    GCTGATCTTAGTTTAGAAAAGGCTGCTTCTGTCCAGTGGGATGAAATGGCTGACATTACG
      A  D  L  S  L  E  K  A  A  S  V  Q  W  D  E  M  A  D  I  T
61   GGTTCATCCCCAATCATTGAGGTTAAACAGGACGAAGATGGATCCTTCTCTATTAGAGAT
      G  S  S  P  I  I  E  V  K  Q  D  E  D  G  S  F  S  I  R  D
121  GTTGAGGAAACCAACATGATAACTCTGGGTGGTGGGGGTTCCGGAGGAGGAGGGTCAGGT
      V  E  E  T  N  M  I  T  L  G  G  G  G  S  G  G  G  G  S  G
181  GCATTATGGGACGTACCCTCTCCCGCAGCCACTCAAAAAGCCACACTAAGTGAGGGTGTC
      A  L  W  D  V  P  S  P  A  A  T  Q  K  A  T  L  S  E  G  V
241  TACAGGATTATGCAACGAGGCTTGTTTGGAAAAACTCAAGTCGGAGTCGGAATACACATG
      Y  R  I  M  Q  R  G  L  F  G  K  T  Q  V  G  V  G  I  H  M
301  GAGGGAGTTTTTCACACAATGTGGCATGTAACAAGAGGTTCTGTTATCTGTCATGAAACC
      E  G  V  F  H  T  M  W  H  V  T  R  G  S  V  I  C  H  E  T
361  GGTCGATTGGAACCTAGTTGGGCTGATGTGAGAAATGACATGATATCTTACGGTGGTGGA
      G  R  L  E  P  S  W  A  D  V  R  N  D  M  I  S  Y  G  G  G
421  TGGCGTCTGGGAGATAAGTGGGACAAAGAGGAGGACGTGCAAGTGTTGGCCATTGAACCA
      W  R  L  G  D  K  W  D  K  E  E  D  V  Q  V  L  A  I  E  P
481  GGCAAAAACCCAAAGCATGTTCAAACCAAGCCTGGACTTTTCAAGACATTGACTGGCGAG
      G  K  N  P  K  H  V  Q  T  K  P  G  L  F  K  T  L  T  G  E
541  ATCGGTGCAGTCACACTTGACTTTAAGCCAGGTACTTCTGGATCACCTATCATTAACAAA
      I  G  A  V  T  L  D  F  K  P  G  T  S  G  S  P  I  I  N  K
601  AAGGGCAAGGTTATAGGTCTGTACGGAAATGGTGTGGTTACCAAATCTGGCGATTATGTT
      K  G  K  V  I  G  L  Y  G  N  G  V  V  T  K  S  G  D  Y  V
661  TCCGCCATTACGCAGGCTGAACGTATCGGGGAACCTGATTATGAAGTAGATGAAGATATC
      S  A  I  T  Q  A  E  R  I  G  E  P  D  Y  E  V  D  E  D  I
721  TTTAGAAAGAAA
      F  R  K  K
```

Figure S4. ^{1}H-NMR data of compound **2** (ChemDiv Catalog number - 6049-2540). [5-((4-chloro-5-methyl-3-nitro-1*H*-pyrazol-1-yl)methyl)-*N*-(1-(2-(diethylamino)ethyl)-1*H*-benzo[d]imidazol-2-yl)furan-2-carboxamide].

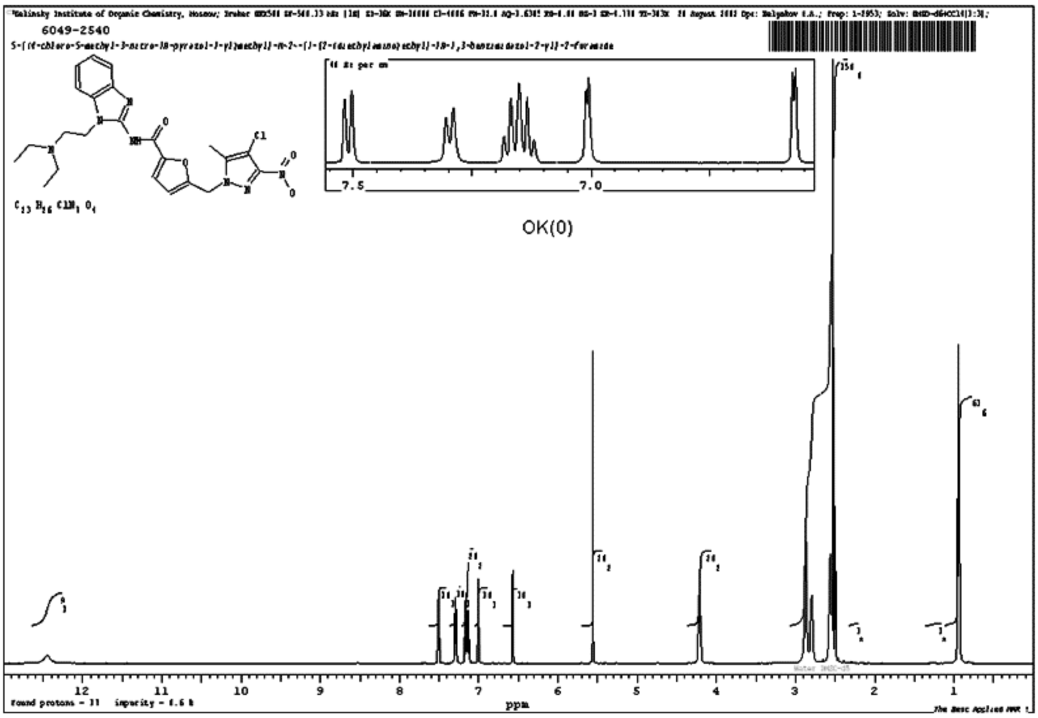

Figure S5. ^{1}H-NMR of compound **5** (Chemdiv ID: E881-0223). 3-((8-benzoyl-1-methyl-[1,2,4]triazolo[4,3-a]quinoxalin-4-yl)amino)benzoic acid.

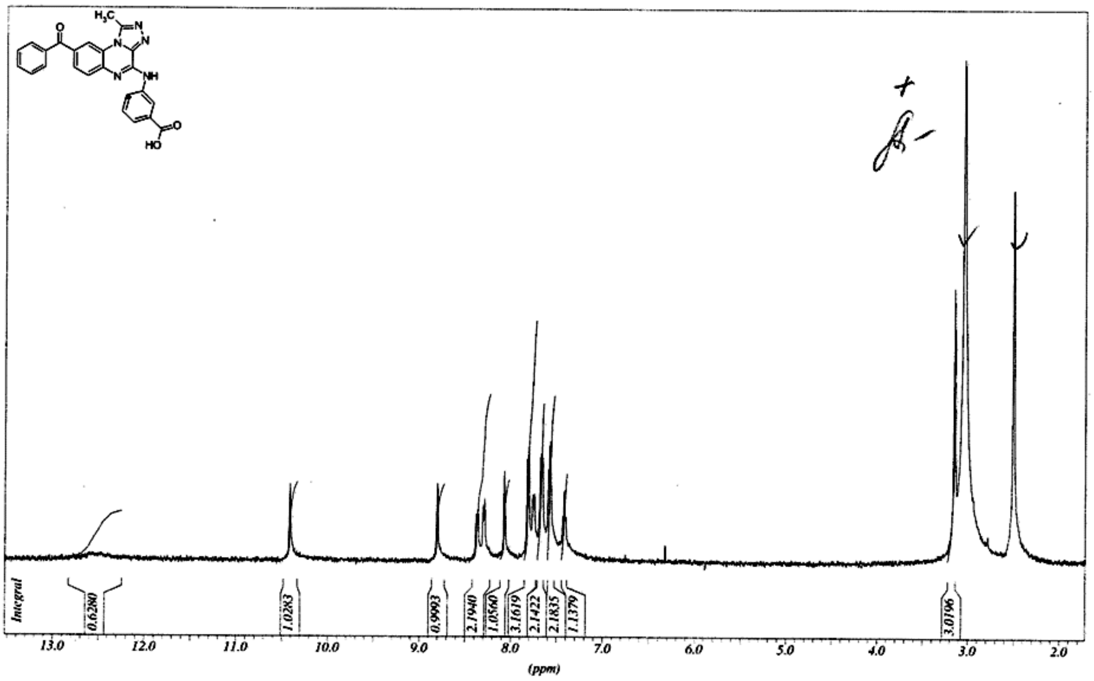

E881- 0223	Oper LV Exp EAV	SF 500.1378	NSC 8	PW 7.00 usec RG 26 SI 16384	Grade OK(00)
Date 30.09.04	Solvent DMSO+CCl_4	SW 7813 Hz	TE 297 K	AQ 1.08 sec RD 9 sec	

Figure S6. ^{1}H-NMR of compound **12** (Chemdiv ID: 3011-0208). 2-(5-(4-fluorophenyl)-3-(thiophen-2-yl)-4,5-dihydro-1*H*-pyrazol-1-yl)-4-(4-(piperidin-1-ylsulfonyl)phenyl)thiazole.

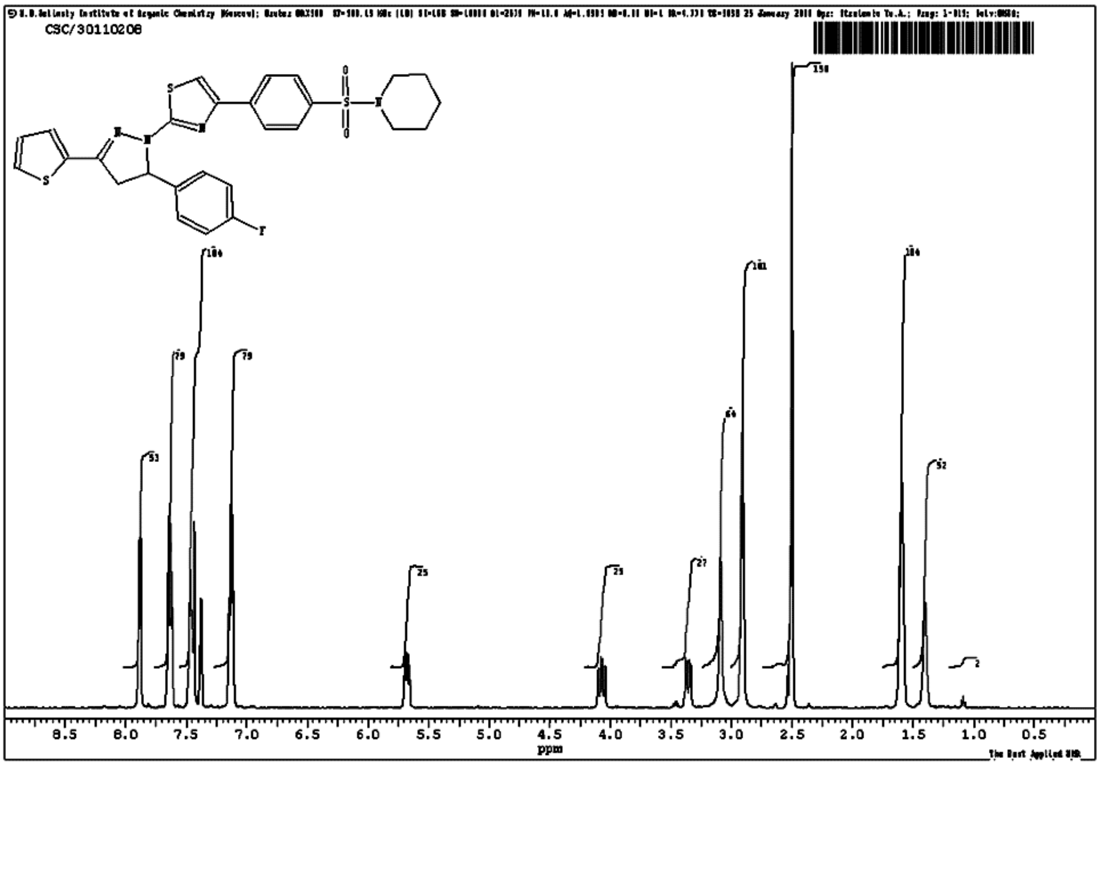

Figure S7. Mass spectrometry of compound **14** (Chemdiv ID: G642-2349). 4-(3-acetyl-5-(2-phenylquinolin-4-yl)-2,3-dihydro-1,3,4-oxadiazol-2-yl)benzoic acid.

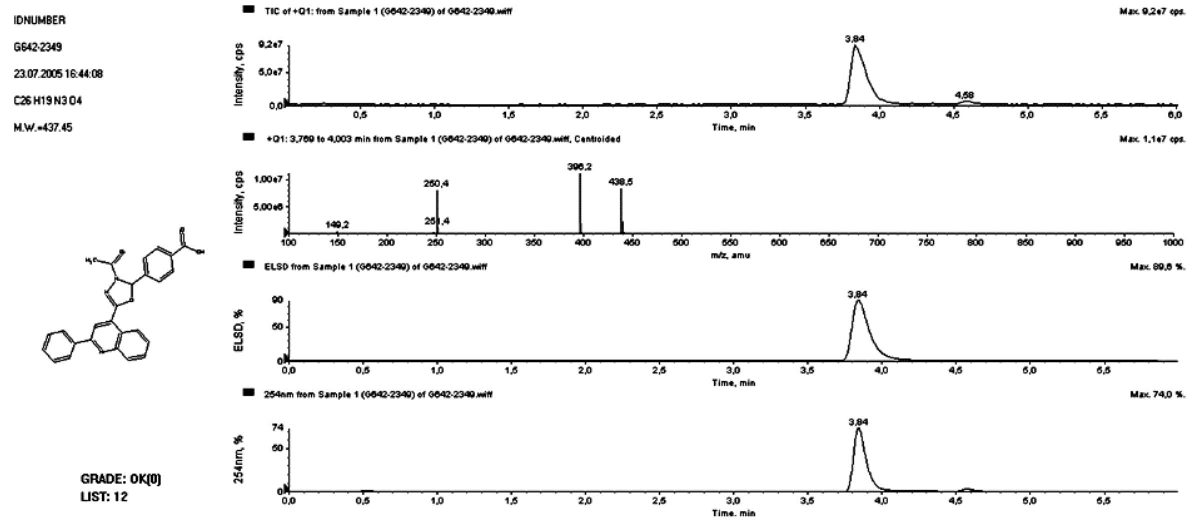

Figure S8. ^{1}H-NMR of compound **22** (Chemdiv ID: K286-0036). 5-[[(7-chloro-4-oxo-4*H*-pyrido[1,2-a]pyrimidin-2-yl)methyl)sulfanyl)-3,6-bis(2,6-dimethylphenyl)-2-thioxo-2,3-dihydro[1,3]thiazolo[4,5-d]pyrimidin-7(6H)-one.

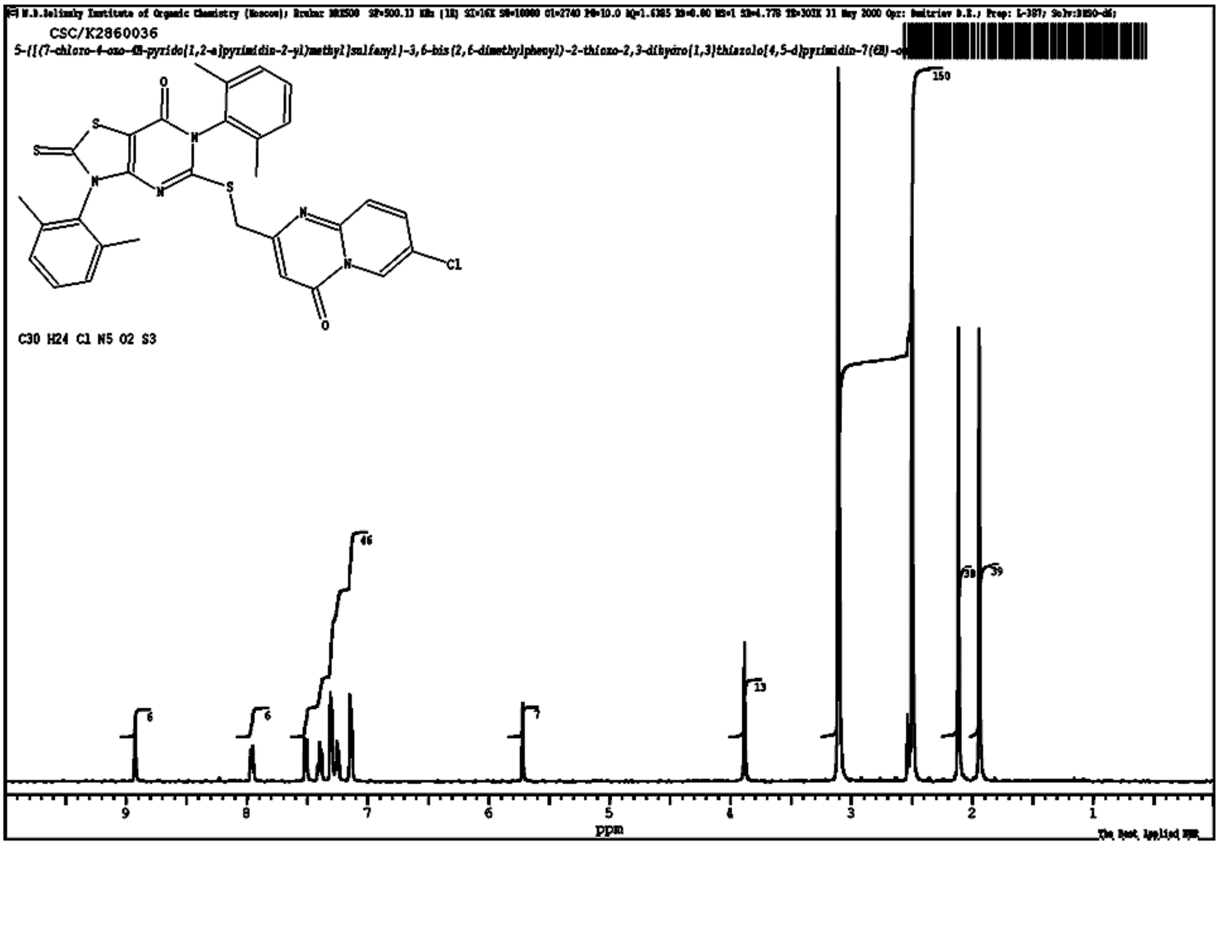

Figure S9. ^{1}H-NMR of compound **27** (Chemdiv ID C090-0497). 4-((3-(azepan-1-yl)-6-oxo-6*H*-anthra[1,9-cd]isoxazol-5-yl)amino)butanoic acid.

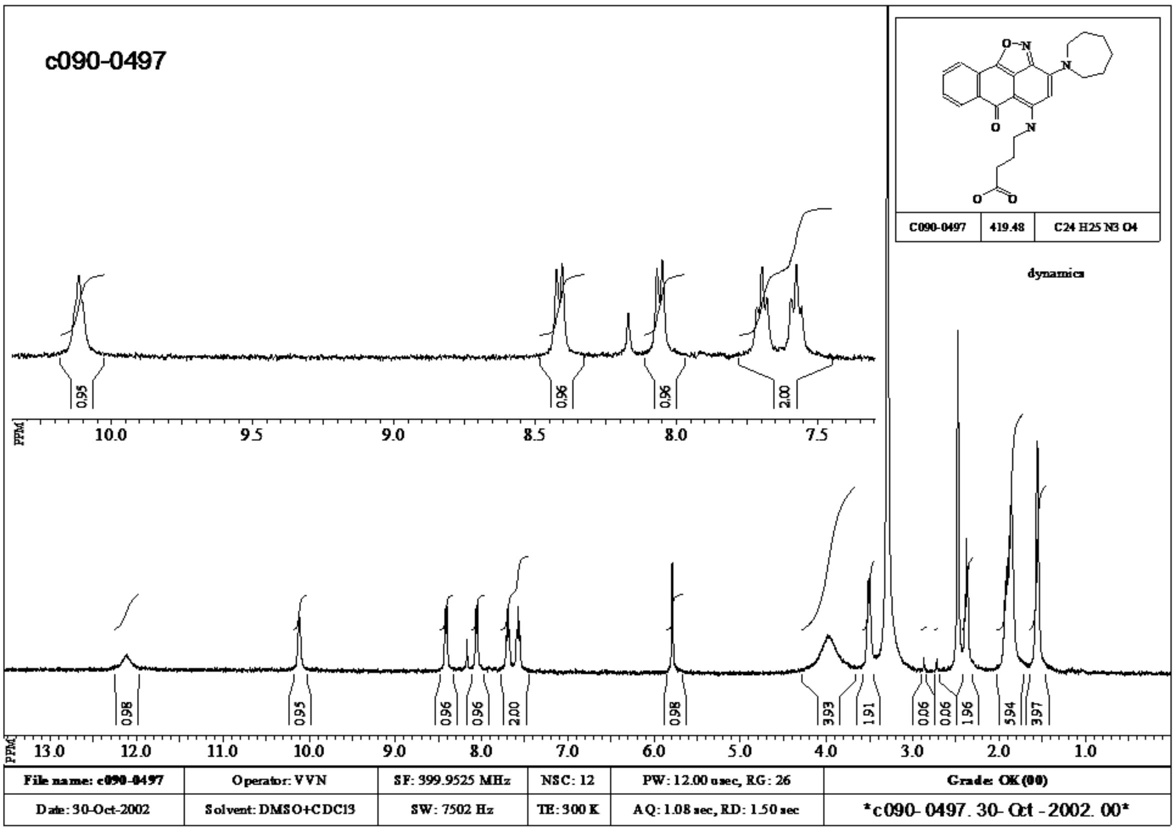

Figure S10. ^{1}H-NMR of compound **29** (Chemdiv ID: F575-0314). 3-((6-(3-fluorophenyl)pyridazin-3-yl)amino)-*N*-(2-(piperidin-1-yl)ethyl)benzamide.

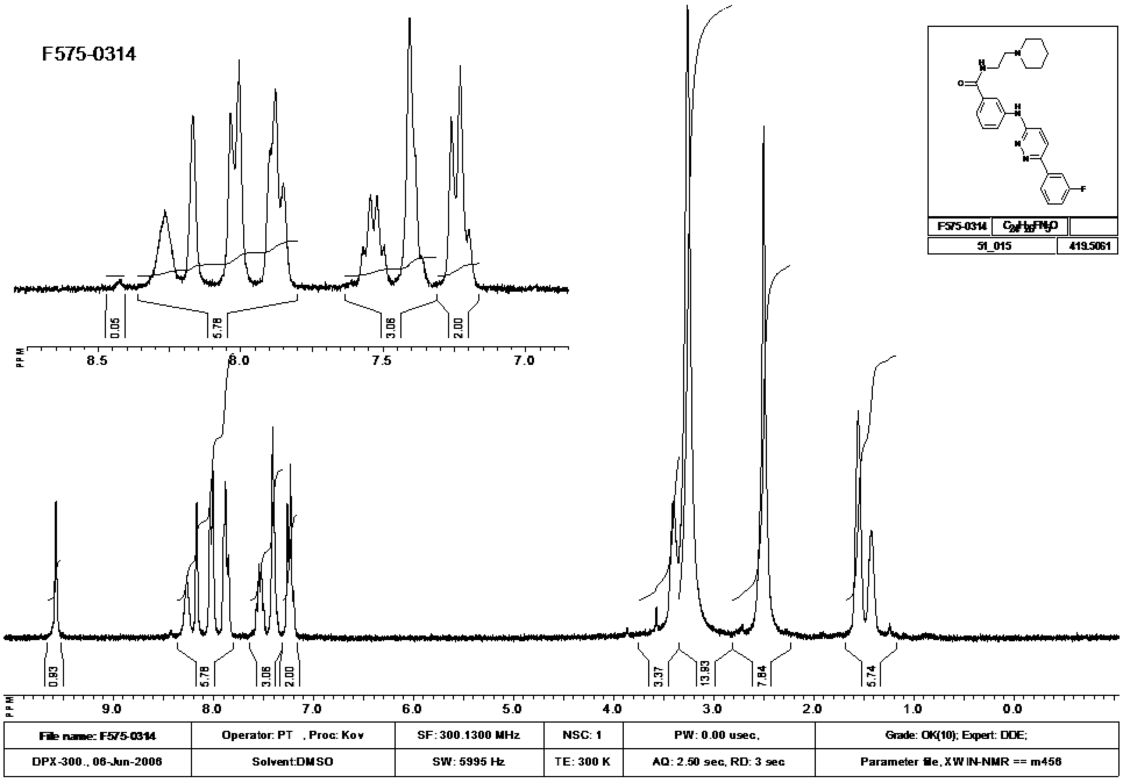

Supplement: Supplementary file 1 [file molecules-18-15600-s001.pdf]
